# “Biomechanical Signaling in Oocytes and Parthenogenetic Cells”

**DOI:** 10.3389/fcell.2021.646945

**Published:** 2021-02-11

**Authors:** Georgia Pennarossa, Fulvio Gandolfi, Tiziana A. L. Brevini

**Affiliations:** ^1^Laboratory of Biomedical Embryology, Department of Health, Animal Science and Food Safety and Center for Stem Cell Research, Università degli Studi di Milano, Milan, Italy; ^2^Laboratory of Biomedical Embryology, Department of Agricultural and Environmental Sciences – Production, Landscape, Agroenergy and Center for Stem Cell Research, Università degli Studi di Milano, Milan, Italy

**Keywords:** oocyte, parthenogenetic cells, hippo signaling pathway, rho GTPase signaling pathway, mechanotransduction

## Abstract

Oocyte-specific competence remains one of the major targets of current research in the field of reproduction. Several mechanisms are involved in meiotic maturation and the molecular signature of an oocyte is considered to reflect its quality and to predict its subsequent developmental and functional capabilities. In the present minireview, we focus on the possible role of mechanotransduction and mechanosensor signaling pathways, namely the Hippo and the RhoGTPase, in the maturing oocyte. Due to the limited access to female gametes, we propose the use of cells isolated from parthenogenetic embryos as a promising model to characterize and dissect the oocyte distinctive molecular signatures, given their exclusive maternal origin. The brief overview here reported suggests a role of the mechanosensing related pathways in oocyte quality and developmental competence and supports the use of uniparental cells as a useful tool for oocyte molecular signature characterization.

## Introduction

It is currently well known that parthenogenesis can spontaneously occur in invertebrates as well as in lower vertebrates. In contrast, in mammals, this type of reproduction is not natural, and the birth of parthenogenetic offspring is not possible. Indeed, in higher vertebrates, with the exception of some avian species, parthenogenesis is usually abortive, because of the requirement of both functionally specialized paternal and maternal genomes that, together with genomic imprinting, act as a developmental barrier (Kono, 2006).

Nevertheless, parthenogenetic activation of mammalian oocytes can be artificially achieved using different *in vitro* approaches that mimic the intracellular calcium wave induced by the spermatozoon at fertilization. This event triggers meiotic resumption with the subsequent cleavage divisions and early embryonic development (Paffoni et al., 2008). However, the resultant parthenotes are not able of developing to term and they arrest growth at different stages, depending on the species (Brevini et al., 2012b), due to the lack of paternally imprinted genes, that are indispensable for the establishment of a functional placenta (Barlow and Bartolomei, 2014).

Parthenogenetic activation allows to circumvent the ethical and legal problems related to the use of human embryos generated for reproductive purposes. Based on this, parthenotes have been employed in different research areas, ranging from assisted reproduction technologies to pluripotent stem cell derivation and basic biology, for studying the regulatory mechanisms underlying the embryonic development (Bos-Mikich et al., 2016). In addition, cell lines obtained from parthenogenetic embryos represent an optimal model to characterize and dissect the distinctive molecular signatures of the oocyte. Indeed, the strictly, and uniquely maternal origin of these cells provides them with the ability to specifically express and/or over-express oocyte-related genes, while offering the possibility to use high-throughput genetic and omics analysis as well as comprehensive studies that require a large quantity of genomic material.

In the present minireview, we focus on the results obtained both in the oocyte and in parthenogenetic cells on the rapidly growing research area of mechanotransduction and suggest its possible role in oocyte quality and developmental competence. In particular, we describe the most relevant mechanosensor signaling pathways, the Hippo and the RhoGTPase, and report their involvement in oocyte and parthenogenetic cell ability to sense and respond to chemical and mechanical cues.

## The Hippo Signaling Pathway and Its Role in Cell Mechanotrasduction

During the last years, increasing evidence has highlighted the importance of biomechanical and biochemical cues deriving from the surrounding cell microenvironment, that play a fundamental role in regulating cell behavior, both in physiological and pathological conditions (Pennarossa et al., 2020a). Cells are indeed able to sense the cellular and extracellular mechanical stimuli and to respond modeling their shape as well as intracellular organization, and modifying their growth, differentiation, and functions. Hence, the origin of the term “mechanotransduction,” which indicates the cellular mechanisms by which mechanical inputs and externally applied forces, such as stretching, contracting, tension or fluid flow, are converted into intracellular signals (Pruitt et al., 2014).

These processes have been shown to be regulated by the highly conserved Hippo signaling pathway that play a fundamental role in controlling cell, tissue and organ development and homeostasis (Camargo et al., 2007). Unlike many other pathways, that are strictly regulated by the binding of specific morphogens and hormones with their corresponding receptors, the Hippo signal responds to a wide range of diverse environmental and physiological cues, including cell-to-cell and cell-to-matrix interactions, mechanical forces, cellular energy status, and various stress stimuli (Wu and Guan, 2020).

The core components of the Hippo pathway consist of a kinase cascade and two main downstream effectors, namely the Yes-associated protein (YAP) and the WW domain-containing transcription regulator protein 1 (WWTR1 or TAZ) (Varelas, 2014). YAP and TAZ compartmentalization depends on the activation or inactivation of the upstream cascade and influences gene expression, controlling cell fate (Brevini et al., 2020). In mammals, upstream regulators include neurofibromin 2 (NF2), mammalian STE20-like protein kinase 1/2 (MST1/2), Salvador family WW domain containing protein 1 (SAV1), MOB kinase activator 1A/B (MOB1A/B), and large tumor suppressor 1/2 (LATS1/2). When stimulated by specific signals, NF2 promotes the activation of the Hippo pathway by interacting with the upstream components MST1/2 and LATS1/2 (Yin et al., 2013). MST1/2 are then activated either by the TAO kinase or through autophosphorylation events (Boggiano et al., 2011; Poon et al., 2011). Subsequently, SAV1 and MOB1A/B, which are regulatory subunits of MST1/2 and LATS1/2, respectively, are phosphorylated by the active form of MST1/2 and recruit LATS1/2 that, in turn, undergo autophosphorylation events (Wu and Guan, 2020). Lastly, activated LATS1/2 phosphorylate YAP and TAZ, leading to their cytoplasmic retention (Zhao et al., 2007). In contrast, when the cascade is inactive, YAP and TAZ translocate into the nucleus, interacting with the other downstream Hippo pathway effectors, namely the DNA-binding protein TEA domain transcription factor 1/2/3/4 (TEAD1–4), and with different transcription factors (TFs). It is noteworthy that YAP and TAZ act as transcriptional coactivators and do not have any DNA-binding domain. Rather, when translocated into the nucleus, they elicit their functions through interaction with other TFs, including TEAD1–4, which are sequence-specific mediators of the Hippo pathway in mammalian cells (Pan, 2010). We recently demonstrated that YAP and TAZ localization was mirrored by a parallel compartmentalization of SMAD2/3 in somatic cells. In particular, we observed that differentiated cells displayed a cytoplasmic retention of YAP and TAZ and exhibited a parallel SMAD2/3 cytoplasmic distribution, while undifferentiated ones showed a nuclear localization of the two molecules, with a concomitant SMAD2/3 nuclear accumulation (Pennarossa et al., 2019, 2020b). Nuclear formation of the YAP/TAZ-SMAD2/3 complex allow binding to TEAD TFs and modulates multiple biological processes, including oogenesis, embryonic development, pluripotency, and immune regulation (Beyer et al., 2013; Mullen, 2014; Huh et al., 2019).

### The Hippo Signaling Pathway in Oocyte

**FIGURE 1:**
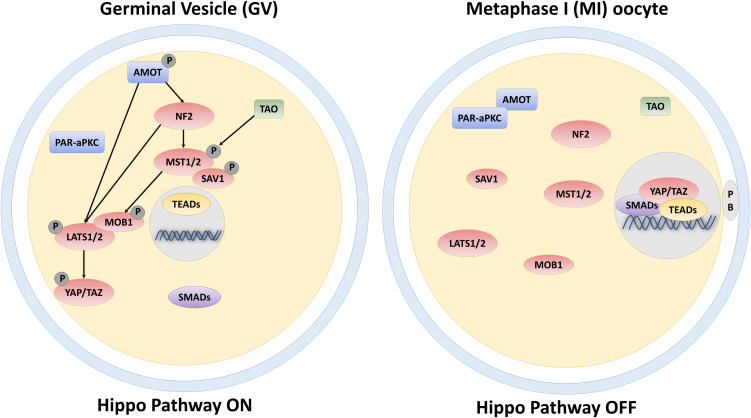
Schematic representation of the core components of the Hippo signaling pathway and their regulation in the GV and MI-stage oocyte.

One of the essential events during oocyte maturation and meiosis is cellular polarization. Indeed, the oocyte undergoes asymmetric meiotic divisions, producing one large cell, the egg, and two small polar bodies. Furthermore, asymmetries are also present in the form of a different compartmentalization of cellular structures and organelles as well as of diverse distribution of proteins and mRNAs within the oocyte. In early-stage oocytes (germinal vesicle, GV), the nucleus is centrally positioned, but, as oogenesis progresses, it moves, and localizes to the cortical area, generating the animal pole. In parallel, a transient aggregation of specific mRNAs, endoplasmic reticulum, mitochondria, Golgi, and proteins, known as the Balbiani body (Guraya, 1979), assembles adjacent to the nucleus, forming the vegetal pole (Marlow, 2010). These two events lead to the establishment of the so-called animal—vegetal axis, necessary for the subsequent organization of the embryonic body axes (Elkouby et al., 2016). Although oocyte polarity is a well-established process, the underlying mechanisms need to be further elucidated and this symmetry breaking is presently suggested to result from biomechanical and biochemical stimuli deriving from cytoskeletal components and specific polarity proteins, respectively. In this direction, different studies demonstrated that proteins involved in controlling polarity, are also able to influence the Hippo pathway activity. In particular, in non-polarized cells, Angiomotin (AMOT) is phosphorylated and activates NF2 and LATS1/2 kinases, facilitating YAP and TAZ phosphorylation and inducing their cytoplasmic retention (Zhao et al., 2011; Li et al., 2015; Figure 1). In contrast, in polarized cells, AMOT is sequestered by the PAR-aPKC system, resulting in YAP and TAZ dephosphorylation, with their consequent nuclear accumulation (Hirate and Sasaki, 2014; Figure 1). In agreement with these observations, immunostaining analysis, carried out on GV oocytes, demonstrated YAP predominant distribution into the cytoplasmic compartment. In contrast, during the subsequent developmental phases, when the oocyte polarizes, YAP protein gradually translocates from the cytoplasm to the nucleus (Yu et al., 2016), suggesting a direct role in maturation and meiosis progression. YAP and TAZ essential role during oogenesis are also suggested by their higher transcription levels compared to somatic cells, both in mouse and human oocytes. Furthermore, a key function of these molecules is also demonstrated by the observation that maternally accumulated YAP plays a key role for zygotic genome activation (Yu et al., 2016) and the subsequent first cell fate decision in the mouse (Nishioka et al., 2009; Frum et al., 2018), and regulates blastocyst development in the porcine (Cao et al., 2019).

### The Hippo Signaling Pathway in Parthenogenetic Cells

The limited number of donors as well as the paucity of the material that may be extracted from each single oocyte, have significantly limited the amount of studies aimed at better elucidating the Hippo signaling pathway in early development. We recently performed a whole transcriptome analysis (WTA) (Pennarossa et al., 2020b), using human parthenogenetic cell lines, previously derived in our laboratory, as a starting material (Brevini et al., 2009, 2012a). Integrative bioinformatics analysis of the omics data obtained revealed that YAP and TAZ genes were differentially expressed in mono-parental cells, compared to bi-parental ones, that were used as a control, with a significant up-regulation of the two genes in parthenogenetic cells (Pennarossa et al., 2020b). This is consistent with the results obtained by Yu et al. (2016) that demonstrated high YAP and TAZ expression in oocytes. We further analyzed the Hippo pathway and the expression of its core components. Interestingly, in contrast to the significantly higher expression levels of the two main actors of the cascade, we could not observe a corresponding increase in the upstream genes, namely LATS1/2, MOB1, MST1/2, NF2, and SAV1, suggesting the possibility of a LATS/MST/NF2-independent regulation of the Hippo pathway. An independent regulatory mechanism has been described in high plasticity cells (Sansores-Garcia et al., 2011; Aragona et al., 2013; Johnson and Halder, 2014) as well as in early developmental stage embryos (Cockburn et al., 2013; Posfai and Rossant, 2016) and appear to be already active in the oocyte. Furthermore, YAP gene upregulation in parthenogenetic cells resulted in an increased synthesis of the encoded protein, that, however, remained in its dephosphorylated form, due to the over described lack of a parallel over expression of LATS1/2, MOB1, MST1/2, NF2, and SAV1 genes, and led to YAP nuclear translocation. This, in turn, allowed for its interaction with the downstream nuclear Hippo pathway effectors, namely TEAD1–4 and SMAD2/3, which were upregulated in parthenogenetic cells, compared to their bi-parental counterparts. These observations are consistent with a recent study carried out in the mouse demonstrating that YAP and TAZ interaction with SMAD2/3 proteins, in the oocyte nucleus, is essential for activating pathways in cumulus and granulosa cells, promoting cell survival and proliferation, while suppressing cell differentiation (Sun and Diaz, 2019).

## The Rho GTPase Signaling Pathway and Its Role in Cell Mechanotrasduction

The Ras homologous (Rho) protein family is a highly conserved member of the Ras superfamily protein and acts as key regulator of several crucial cellular activities, including morphology, migration, and cytokinesis. This protein family plays an essential role in actin reorganization, controlling the dynamic processes of polymerization and depolymerization of contractile actin stress fibers (Ridley and Hall, 1992), and is involved in microtubule organization, controlling their stability, elongation, and alignment (Wojnacki et al., 2014). GTPases are monomeric proteins and function as molecular switches that turn “on” or “off” signal transduction pathways, in response to guanine nucleotide exchange factors (GEF, activators) or GTPase-activating proteins (GAP, inhibitors), respectively (Warner et al., 2019).

Several recent studies demonstrated that Rho GTPases are also essential modulators of the Hippo pathway components in an actin cytoskeleton-dependent manner (Seo and Kim, 2018; Rausch and Hansen, 2020). Early observations revealed that YAP and TAZ were active in cells undergoing spreading and inactive in round and compact cells. Furthermore, they showed YAP and TAZ ability to sense and respond to different stimuli, such as stiffness (Dupont et al., 2011), detachment and attachment proprieties (Zhao et al., 2012), indicating that actin rearrangement may be associated with changes in the Hippo pathway activity. Moreover, both Dupont et al. (2011) and Zhao et al. (2012) demonstrated that the Ras Homolog Family Member A (RhoA) strongly enhances YAP and TAZ activity as well as Rac Family Small GTPase (Rac) and Cell Division Cycle 42 (Cdc42), although less potently. All these observations point to a possible role of Rho GTPases in regulating dynamics of the cytoskeletal actin (Jaffe and Hall, 2005) and strongly suggest that the same actin may act as a mediator and integrator of various upstream signals of the Hippo pathway. In agreement with this hypothesis, it was subsequently demonstrated that alteration of actin dynamics severely affected YAP and TAZ activity. For example, the induction of filamentous actin (F-actin) bundling promoted nuclear enrichment of the two proteins (Aragona et al., 2013; Matsui and Lai, 2013; Yu and Guan, 2013), while F-actin-disrupting caused their cytoplasmic retention (Wada et al., 2011; Zhao et al., 2012). Although the specific mechanisms involved still remain unclear, it is well known that Rho GTPases activities enhance actin polymerization, inducing the formation of stress fibers (Sadok and Marshall, 2014) and leading to the production of two different mechanotransduction effects, described as short- or long-range transmission of force (Miroshnikova et al., 2017). The first directly affects nuclear localization and function of mechanosensitive transcription regulators, including YAP and TAZ. The long-range effect transmits the force from the surrounding environment to the cell nucleus, thanks to a physical actin-mediated connection with the linker of nucleoskeleton and cytoskeleton (LINC) complex. This latter event generates several changes, including chromatin remodeling, exposure of DNA specific sites to TFs, and the modifications of nuclear pore conformation and size, eventually promoting YAP and TAZ nuclear translocation.

### The Rho GTPase Signaling Pathway in Oocyte

As described above, mammalian oocyte maturation involves a unique asymmetric division that is controlled by microtubules and cytoskeletal actin (Sun et al., 2001; Brunet and Verlhac, 2011). Full-grown oocytes arrested at the GV stage resume meiosis after germinal vesicle breakdown and a spindle is organized at or near the center of the cytoplasm. After chromosomes align in the equatorial plate of the oocyte, the spindle migrates, in an actin-dependent manner, along its long axis, and toward the cortex. In addition, an event known as “cortical reorganization” takes place, during which cortical granules are redistributed, actin becomes enriched to form an actin cap, and microvilli are lost in the region overlying the spindle (Deng et al., 2003). Subsequently, thanks to the formation of an actomyosin-based contractile ring, cytokinesis occurs, leading to the extrusion of the first polar body (Kutsuna et al., 2004).

As expected, several molecules have been reported to be involved in these complex processes. Among them the Rho GTPases play a fundamental role, as coordinators of actin filament and microtubule formation and dynamics (Schmandke et al., 2007). Recent studies showed that this protein family is necessary for polar body extrusion and spindle rotation during meiosis (Duan and Sun, 2019). In particular, it was demonstrated that RhoA accumulates at the contractile ring, during the late phases of meiosis, and its inhibition or knockdown cause aberrant actin assembly and failure of polar body extrusion, both in mouse and porcine oocytes (Zhong et al., 2005; Zhang et al., 2008, 2014). Similarly, two other members of the Rho GTPase family, namely Cdc42, and Rac, play a key role during oocyte maturation. Indeed, their mutation causes aberrant cortical polarity establishment (Cui et al., 2007; Bielak-Zmijewska et al., 2008; Wanga et al., 2013) and failure of chromosome alignment, resulting in altered polar body extrusion (Song et al., 2016; Hao et al., 2017).

### The Rho GTPase Signaling Pathway in Parthenogenetic Cells

In agreement with the data obtained in the female gamete, human parthenogenetic cells displayed higher expression of the Rho GTPase family components, namely RHOA, RHOB, and RHOC, compared to bi-parental ones. Furthermore, a down-regulation of 8 out of 11 GAP inhibitors and a higher transcription levels for 12 out of 17 of GEF activators were detected in mono-parental cells (Pennarossa et al., 2020b).

Altogether, these results indicate that, both in oocytes and in parthenogenetic cells, a fine-tuning system is likely to regulate mechanotrasduction and mechanosensing pathways in a dual and cooperating mode: a direct regulation exerted through the Hippo signaling and an indirect control mediated via the expression of the Rho GTPase activators and inhibitors.

## Conclusion

**FIGURE 2:**
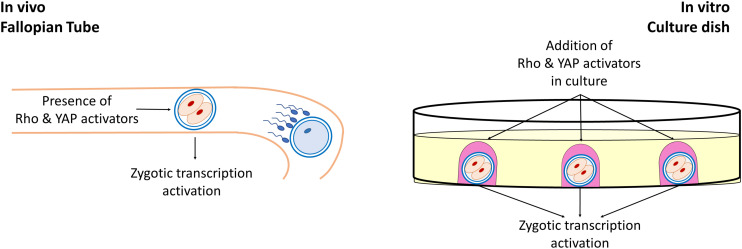
Role of Rho and YAP activators in early embryonic development. During early development of mammalian embryos, Rho, and YAP activators present in the tubal fluid induce the activation of the Hippo and RhoGTPase signaling pathways, playing an essential for the zygotic genome activation. Similarly, during *in vitro* embryo culture, the addition of exogenous Rho and YAP activators in the culture medium exerts a beneficial effect in early embryonic development.

Biomechanical cues deriving from the surrounding cell microenvironment play a key role in several experimental, settings and act, together with the biochemical components, in regulating cell behavior, in physiological and pathological conditions. Cells are able to sense the cellular and extracellular mechanical stimuli and to respond, modeling shape and intracellular organization, modifying their growth, differentiation, and function. This ability is not limited to somatic cells and has emerged as an active property of oocytes and early embryos, that may actively sense biomechanical stimuli and convert them into intracellular signals, tuning their own behavior. In the female gamete context, the two main mechanosensing signaling pathways, namely the Hippo and the RhoGTPase, have been demonstrated to be involved in many fundamental oogenesis processes and to influence oocyte quality. While the RhoGTPase signaling pathways are important actors in the coordination of actin filaments and microtubule formation as well as polar body extrusion and spindle rotation during meiosis, the main actors of the Hippo pathway, YAP and TAZ have an essential role during oogenesis, are highly transcribed in mouse and human oocytes, are maternally accumulated and have been reported as strong candidates for zygotic genome activation (Figure 2).

It is clear that our current understanding of how these complex and dynamic mechanisms are implicated throughout the course of oocyte maturation is far from complete. A heavy limit is represented by the paucity of the material obtained from each oocyte, that makes it difficult to carry out high-throughput and omics studies. Some of the results summarized in this review were therefore obtained in cell lines generated from parthenogenetic embryos, that may constitute an alternative and particularly advantageous model, since they allow to circumvent the problems related to the little amount of material available, while, at the same time, reflecting the distinctive molecular signatures of the oocyte.

In conclusion, the data presently available, although still very much in need of further elucidation, suggest a role of cellular mechanics and mechanotransduction in mammalian female gamete maturation, ultimately affecting oocyte quality and competence to develop in a healthy embryo.

## Author Contributions

GP drafted the manuscript. FG designed and coordinated the study and drafted the manuscript. TB designed and coordinated the study and drafted the manuscript. All authors contributed to the article and approved the submitted version.

## Conflict of Interest

The authors declare that the research was conducted in the absence of any commercial or financial relationships that could be construed as a potential conflict of interest.
